# Supplementary material for: Low Self-Perceived Cooking Skills Are Linked to Greater Ultra-Processed Food Consumption Among Adolescents: The EHDLA Study
Source: Nutrients. 2025 Mar 28;17(7):1168. doi: 10.3390/nu17071168 (PMC11990398; doi:10.3390/nu17071168)
Supplement: Supplementary file 1 [file nutrients-17-01168-s001.zip › nutrients-3522920 - supplementary.pdf]

Supplementary materials

Table S1. Robust linear model examining the association of self-perceived cooking skills and servings of ultra-processed food consumed (and covariates) in adolescents.

Predictor	*B*	95% CI	*p* value
Self-perceived cooking skills			
Very inadequate	Reference		
Inadequate	-1.71	-3.69, 0.26	0.089
Adequate	-2.00	-3.84, -0.16	0.033
Very adequate	-3.28	-5.40, -1.15	0.003
Age (per year)	-1.08	-1.45, -0.71	<0.001
Sex			
Boys	Reference		
Girls	-0.92	-2.06, 0.23	0.117
FAS-III (per point)	-0.39	-0.65, -0.14	0.002
Overall sleep duration (per hour)	-0.01	-0.02, 0.00	0.093
YAP-S physical activity (per point)	-1.06	-1.90, -0.22	0.013
YAP-S sedentary behaviors (per point)	1.96	1.02, 2.91	<0.001
Energy intake (per 1000 kcal)	0.01	0.01, 0.01	<0.001
BMI (per kg/m^2)	-0.15	-0.27, -0.04	0.010

B, unstandardized beta coefficient; BMI, body mass index; CI, confidence interval; FAS-III, Family Affluence Scale-III; YAP-S, Spanish Youth Activity Profile.

Table S2. Robust linear model examining the associations between self-perceived cooking skills and servings of sausages consumed (and covariates) in adolescents.

Predictor	*B*	95% CI	*p* value
Self-perceived cooking skills			
Very inadequate	Reference		
Inadequate	0.03	-0.58, 0.65	0.917
Adequate	-0.24	-0.81, 0.33	0.409
Very adequate	-0.59	-1.25, 0.07	0.079
Age (per year)	0.05	-0.07, 0.17	0.394
Sex			
Boys	Reference		
Girls	0.23	-0.13, 0.59	0.212
FAS-III (per point)	0.08	0.00, 0.16	0.050
Overall sleep duration (per hour)	0.00	0.00, 0.00	0.734
YAP-S physical activity (per point)	-0.25	-0.52, 0.01	0.058
YAP-S sedentary behaviors (per point)	-0.19	-0.48, 0.11	0.210
Energy intake (per 1000 kcal)	0.00	0.00, 0.00	<0.001
BMI (per kg/m^2)	0.01	-0.02, 0.05	0.454

B, unstandardized beta coefficient; BMI, body mass index; CI, confidence interval; FAS-III, Family Affluence Scale-III; YAP-S, Spanish Youth Activity Profile.

Table S3. Robust linear model examining the association of self-perceived cooking skills and servings of fast food consumed (and covariates) in adolescents.

Predictor	*B*	95% CI	*p* value
Self-perceived cooking skills			
Very inadequate	Reference		
Inadequate	0.36	-0.07, 0.80	0.105
Adequate	0.43	0.03, 0.84	0.037

Predictor	B	95% CI	p value
Very adequate	0.37	-0.10, 0.84	0.120
Age (per year)	-0.09	-0.17, -0.01	0.036
Sex			
Boys	Reference		
Girls	0.13	-0.12, 0.38	0.316
FAS-III (per point)	-0.01	-0.07, 0.04	0.692
Overall sleep duration (per hour)	0.00	0.00, 0.00	0.626
YAP-S physical activity (per point)	-0.06	-0.25, 0.12	0.511
YAP-S sedentary behaviors (per point)	0.08	-0.13, 0.29	0.457
Energy intake (per 1000 kcal)	0.00	0.00, 0.00	<0.001
BMI (per kg/m²)	0.01	-0.02, 0.04	0.456

B, unstandardized beta coefficient; BMI, body mass index; CI, confidence interval; FAS-III, Family Affluence Scale-III; YAP-S, Spanish Youth Activity Profile.

Table S4. Robust linear model examining the associations between self-perceived cooking skills and servings of dairy products consumed (and covariates) in adolescents.

Predictor	B	95% CI	p value
Self-perceived cooking skills			
Very inadequate	Reference		
Inadequate	-0.58	-1.23, 0.06	0.078
Adequate	-0.46	-1.06, 0.15	0.138
Very adequate	-0.84	-1.53, -0.14	0.019
Age (per year)	-0.22	-0.34, -0.10	<0.001
Sex			
Boys	Reference		
Girls	-0.60	-0.97, -0.22	0.002
FAS-III (per point)	-0.09	-0.18, -0.01	0.028
Overall sleep duration (per hour)	0.00	0.00, 0.00	0.490
YAP-S physical activity (per point)	-0.09	-0.37, 0.18	0.515
YAP-S sedentary behaviors (per point)	0.12	-0.19, 0.43	0.448
Energy intake (per 1000 kcal)	0.00	0.00, 0.00	<0.001
BMI (per kg/m²)	-0.02	-0.06, 0.01	0.211

B, unstandardized beta coefficient; BMI, body mass index; CI, confidence interval; FAS-III, Family Affluence Scale-III; YAP-S, Spanish Youth Activity Profile.

Table S5. Robust linear model examining the associations between self-perceived cooking skills and servings of beverages consumed (and covariates) in adolescents.

Predictor	B	95% CI	p value
Self-perceived cooking skills			
Very inadequate	Reference		
Inadequate	-0.65	-1.56, 0.26	0.164
Adequate	-0.85	-1.71, 0.00	0.052
Very adequate	-0.67	-1.65, 0.32	0.185
Age (per year)	-0.18	-0.36, -0.01	0.036
Sex			
Boys	Reference		
Girls	-0.46	-0.99, 0.07	0.091
FAS-III (per point)	-0.26	-0.37, -0.14	<0.001
Overall sleep duration (per hour)	0.00	-0.01, 0.00	0.813
YAP-S physical activity (per point)	0.06	-0.33, 0.45	0.769
YAP-S sedentary behaviors (per point)	0.58	0.14, 1.02	0.010

Predictor	B	95% CI	p value
Energy intake (per 1000 kcal)	0.00	0.00, 0.00	<0.001
BMI (per kg/m^2)	0.02	-0.04, 0.07	0.580

B, unstandardized beta coefficient; BMI, body mass index; CI, confidence interval; FAS-III, Family Affluence Scale-III; YAP-S, Spanish Youth Activity Profile.

Table S6. Robust linear model examining the associations between self-perceived cooking skills and servings of beverages consumed (and covariates) in adolescents.

Predictor	B	95% CI	p value
Self-perceived cooking skills			
Very inadequate	Reference		
Inadequate	0.05	-0.28, 0.39	0.747
Adequate	0.10	-0.21, 0.41	0.525
Very adequate	-0.17	-0.52, 0.19	0.362
Age (per year)	-0.07	-0.13, 0.00	0.036
Sex			
Boys	Reference		
Girls	0.29	0.10, 0.48	0.003
FAS-III (per point)	-0.02	-0.07, 0.02	0.255
Overall sleep duration (per hour)	0.00	0.00, 0.00	0.256
YAP-S physical activity (per point)	-0.05	-0.19, 0.09	0.453
YAP-S sedentary behaviors (per point)	0.32	0.16, 0.48	<0.001
Energy intake (per 1000 kcal)	0.00	0.00, 0.00	<0.001
BMI (per kg/m^2)	-0.02	-0.04, 0.00	0.113

B, unstandardized beta coefficient; BMI, body mass index; CI, confidence interval; FAS-III, Family Affluence Scale-III; YAP-S, Spanish Youth Activity Profile.

Table S7. Robust linear model examining the associations between self-perceived cooking skills and servings of beverages consumed (and covariates) in adolescents.

Predictor	B	95% CI	p value
Self-perceived cooking skills			
Very inadequate	Reference		
Inadequate	-0.25	-1.55, 1.04	0.700
Adequate	-0.42	-1.63, 0.78	0.490
Very adequate	-1.73	-3.12, -0.33	0.016
Age (per year)	-0.62	-0.86, -0.37	<0.001
Sex			
Boys	Reference		
Girls	0.27	-0.48, 1.02	0.483
FAS-III (per point)	-0.12	-0.28, 0.05	0.176
Overall sleep duration (per hour)	0.00	-0.01, 0.01	0.767
YAP-S physical activity (per point)	-0.59	-1.14, -0.04	0.034
YAP-S sedentary behaviors (per point)	1.18	0.56, 1.80	<0.001
Energy intake (per 1000 kcal)	0.00	0.00, 0.00	<0.001
BMI (per kg/m^2)	-0.13	-0.21, -0.05	0.001

B, unstandardized beta coefficient; BMI, body mass index; CI, confidence interval; FAS-III, Family Affluence Scale-III; YAP-S, Spanish Youth Activity Profile.
